# Genes associated with genetic and rare lung diseases and the risk of lung cancer

**DOI:** 10.21203/rs.3.rs-7029929/v1

**Published:** 2025-08-11

**Authors:** Albert Rosenberger, Heike Bickeböller, David C Christiani, Neil E. Caporaso, Geoffrey Liu, Stig E. Bojesen, Loic Le Marchand, Demetrios Albanes, Melinda C. Aldrich, Adonina Tardon, Guillermo Fernández-Tardón, Gad Rennert, John K. Field, Michael P. A. Davies, Lambertus A. Kiemeney, Philip Lazarus, Shanbeh Zienolddiny, Stephen Lam, Matthew B. Schabath, Angeline S. Andrew, Susanne M Arnold, Gary E. Goodman, Jennifer A. Doherty, Fiona Taylor, Angela Cox, Penella J. Woll, Angela Risch, Mikael Johansson, Paul Brennan, Maria Teresa Landi, Sanjay S. Shete, Rayjean J Hung, Christopher I. Amos, The INTEGRAL-ILCCO Consortium

**Affiliations:** a. Department of Genetic Epidemiology, University Medical Center, GeorgAugust-University Göttingen, Göttingen, Germany.; b. Department of Environmental Health, Harvard T.H. Chan School of Public Health and Massachusetts General Hospital/Harvard Medical School, Boston, MA, USA.; c. Division of Cancer Epidemiology and Genetics, National Cancer Institute, US National Institutes of Health, Bethesda, MD, USA.; d. Medical Oncology and Medical Biophysics, Princess Margaret Cancer Centre, Toronto, ON, Canada.; e. Medicine and Epidemiology, Dalla Lana School of Public Health, University of Toronto, Toronto, ON, Canada.; f. Department of Clinical Biochemistry, Herlev and Gentofte Hospital, Copenhagen University Hospital, Copenhagen, Denmark.; g. Faculty of Health and Medical Sciences, University of Copenhagen, Copenhagen, Denmark.; h. Copenhagen General Population Study, Herlev and Gentofte Hospital, Copenhagen, Denmark.; i. Epidemiology Program, University of Hawaii Cancer Center, Honolulu, HI, USA.; j. Department of Thoracic Surgery, Division of Epidemiology, Vanderbilt University Medical Center, Nashville, TN, USA.; k. Health Research Instotute of Asturias (ISPA) and University Nebrija, Asturias, Spain.; l. Clalit National Cancer Control Center at Carmel Medical Center and Technion Faculty of Medicine, Haifa, Israel.; m. Department of Molecular and Clinical Cancer Medicine, Roy Castle Lung Cancer Research Programme, The University of Liverpool, Liverpool, UK.; n. Departments of IQ Health and Urology, Radboud University Medical Center, Nijmegen, The Netherlands.; o. Department of Pharmaceutical Sciences, College of Pharmacy, Washington State University, Spokane, WA, USA.; p. National Institute of Occupational Health, Oslo, Norway.; q. Department of Integrative Oncology, University of British Columbia, Vancouver, British Columbia, Canada.; r. Department of Cancer Epidemiology, H. Lee Moffitt Cancer Center and Research Institute, Tampa, FL, USA.; s. Department of Epidemiology, Geisel School of Medicine, Hanover, NH, USA.; t. Markey Cancer Center, University of Kentucky, Lexington, KY, USA.; u. Swedish Medical Group, Seattle, WA, USA.; v. Department of Population Health Sciences, Huntsman Cancer Institute, University of Utah, Salt Lake City, UT, USA.; w. Department of Oncology and Metabolism, University of Sheffield, Sheffield, UK.; x. University of Salzburg and Cancer Cluster Salzburg, Salzburg, Austria.; y. Member of the German Center for Lung Research (DZL), Translational Lung Research Center (TLRC) Heidelberg, Heidelberg, Germany.; z. Translational Research Unit, Thoraxklinik, University Hospital Heidelberg, Heidelberg, Germany.; aa. Department of Radiation Sciences, Umeå University, Umeå, Sweden.; bb. International Agency for Research on Cancer, World Health Organization, Lyon, France.; cc. Department of Biostatistics, Division of Basic Sciences, The University of Texas MD Anderson Cancer Center, Houston, TX, USA.; dd. The University of New Mexico Comprehensive Cancer Center, Albuquerque, New Mexico, USA.; ee. Lunenfeld-Tanenbaum Research Institute, Sinai Health System, University of Toronto, Toronto, Ontario, Canada.; ff. Dalla Lana School of Public Health, University of Toronto, Toronto, Canada

**Keywords:** lung cancer, genomic marker, rare disease, GARD, gene-set analysis, gene-based test, adrenocorticotropic hormone, Hypothalamic-Pituitary-Adrenal axis, OMIM, Orphanet, CYP2A6, DMD, LTBP4

## Abstract

**Background:**

We investigated whether markers, genes or terms of the *Human Phenotype Ontology* associated with genetic or rare diseases (GARDs) that affect airway or lung function are associated with lung cancer.

**Methods:**

Genes of interest were extracted from *GARD*, *OMIM*, *ORPHANET* and Monarch Initiative. Individual SNP, gene level and gene-set analyses were performed for 52,207 SNPs, 1,677 genes or for 620 terms of the *Human Phenotype Ontology*. The analysis included 14,068 lung cancer cases and 12,390 cancer-free control subjects of European descent from the International Lung Cancer Consortium ILCCO.

**Results:**

The marker rs56113850 (OR=0.893, 95%CI: 0.862–0.924) was associated with lung cancer (p=1.2×10^−10^). This marker is located in CYP2A6 as well as in an enhancer region of *LTBP4*, which is associated with cutis laxa. A suggestive significant association was observed for two markers associated with the *DMD* gene, which is linked to Duchenne muscular dystrophy. The gene sets “Abnormal circulating adrenocorticotropin concentration” and “Central nervous system neoplasm” were found to be significantly enriched with GARD genes, and can therefore be considered to be associated with lung cancer.

**Conclusions:**

Genes associated with genetic and rare lung diseases do not generally appear to carry risk factors for lung cancer. However, genes associated with the hypothalamic-pituitary-adrenal axis show some, but rather weak or complex, associations with lung cancer. Tests at the gene level provide extremely inhomogeneous results, even when applied to the same data.

## Introduction

Lung cancer (LC) is one of the most common and deadliest cancers worldwide. It is the second most common cancer in men and the third most common cancer in women. [1] However, LC incidence varies considerably by geographical region and sex. [2] Five-year survival remains low at 13–18%. [3] Smoking is the predominant but not the only known risk factor. Other important factors that increase the risk of lung cancer are exposure to harmful substances, asbestos, radon and other chemicals, as well as diseases such as pneumonia, emphysema, pulmonary fibrosis and others. [1]

Over the last three decades, numerous genomic loci have been identified that confer an individual genetic predisposition to LC. As part of large-scale genome-wide association studies (GWAS), individual variants have been found in genes/regions such as *CHRNA5/CHRNA3/CHRNB4* on 15q25.1, *TERT/ CLPTM1L* on 5p15.33, *MSH5/BAG6* on 6p21.33, *CHEK2* on 22q12.1 and many others that co-determine susceptibility to LC. [4] The genetic landscape of susceptibility differs to some extent between Europeans and Asians, men and women, or smokers and non-smokers. [5, 6] For example, some of the strongest lung cancer susceptibility variants on 15q25, identified in Europeans, have very low allele frequencies in Asian populations, or conversely, variants on 6p21.

As thousands of markers are tested in GWAS, appropriate significance levels were set to minimise spurious associations. The following definitions were proposed: 1) suggestive association, where one false-positive association is expected per GWAS, and 2) genome-wide significant association, where one false-positive association is expected to occur 0.05 times per GWAS. [7] Genome-wide significance limits range between about 1×10^−7^ and 3×10^−8^ depending on the genetic population investigated. [8] Suggestive significance is often considerated if p-values are lower than e.g 5×10^−6^ or 5×10^−4^.[9–11] As these significance levels of GWAS are very low, many true associated markers/genes remain undetected, even if the sample consists of several thousand cases and controls and the p-values are below 0.05. Some post-GWAS methods have been developed to detect genomic loci or genes associated with a trait in these grey areas. [11] E.g. complex interaction of MTAB and DKK2 were found associated to lung cancer within never smokers by gene-set (pathway-based) analyses. [12] Several methods and computer routines to perform multi-marker tests are available to enable SNP-based and gene-based association tests. [13–16]

More than 7000 types of rare diseases, each affecting less than one in 2000 people, exist. Approximately 300 million people live with rare diseases. Thus their worldwide burden is significant. Around 80% of rare diseases have a genetic cause. [17] Several public available sources provide information on rare and genetic diseases, as the *Genetic and Rare Diseases Information Center* (*GARD*, [18]) established by the US National Institutes of Health (NIH), the *OMIM database* (Online Mendelian Inheritance in Man^®^, [19]), the *ORPHANET* (Knowledge on rare diseases and orphan drugs, [20]) or the *Monarch Initiative*. [21] These and other data bases have been consolidated and harmonised in recent years. Data entries were further linked to the *Human Phenotype Ontology* (HPO, [22]).

Many of these rare and genetic diseases affect the respiratory system or are categorised as cancers. We therefore investigated whether markers, genes or HPO terms associated with genetic or rare diseases that affect the airways or lung function are associated with lung cancer within the large-scale series of cases and controls of European descent held by the International Lung Cancer Consortium (ILCCO) / Integrative analysis of Lung Cancer Etiology and Risk (INTEGRAL).

## Methods

The work presented has been reviewed and approved by the ILCCO Steering Committee.

### Cases and Controls

We used phenotype and genotype data of 58,181 entries of the data repository of ILCCO. Details of the repository were described previously. [23, 24] DNA samples were genotyped with the Illumina Infinium OncoArray-500K. Genotype imputation was performed for all subjects in the cohort by using 32,470 reference samples from the Haplotype Reference Consortium. Individuals without information on smoking status or age, and samples of poor genotyping quality or sex discrepancies, were left out. Low-quality variants were filtered out. [25] To avoid population stratification, this analysis is focused on European-ancestry population (defined as more than 95% probability of being of European descent). 14,068 incident LC cases and 12,390 cancer-free controls of European descent remained for analysis.

### HPO Classification

We retrieved all the “terms” of the *Human Phenotype Ontology* (HPO) [22] and organized them according to the tree structure (levels) of the ontology. We traced and ordered each term back to its parent and grandparent and so on up to the level-1 term “HP:0000001 All”. From this, we created a list of all 979 terms from levels 2 to 6, which we reduced to 620 HPO terms of interest describing phenotypes of the respiratory system, cardiovascular system, nerves or brain, immune system including blood components, infections, inflammation or cancer.

### Selection genes from genetic or rare diseases

We have brought together information of the rare diseases contained in *GARD*, *OMIM*, *ORPHANET* and *Monarch Initiative* and extracted all the genes linked to them. The data was downloaded no later than January 2023. The gene names were cross-checked against the common nomenclature of tgeo HGNC [26] to avoid ambiguities. We linked all genes with HPO terms, where HPO identifiers or linkings were provided by *GARD*, *ORPHANET* or *OMIM*. Next, 20 genes of the HLA-region, 42 genes located in log-LD regions, 279 immuno-related genes and 50 genes with known or more intensly investigated associations to LC were left out. We ended up with 1,677 genes included in the analysis.

### Variant to gene assignment and SNP filtering

We harmonized all positions to genome reference build hg37 applying *hgLiftOver* as necessary. Next, in total 58,836 SNPs available in the investigated ILCCO sample were assigned to genes if a) located within a gene (between start codon and end codon), b) located 500bp down- and upstream of the gene to cover promoter and termination regions, or c) located in a related enhancer regions. Enhancer regions were defined according to GeneHancer 4.4. (as of 2017; gene-enhancer-score >0.5) or the EnhancerAtlas 2.0 (for tissue/cell type Lung and Bronchia_epithelial) or Super Enhancer Archive (SEA Version 3.0, Tissues: lung_fibroblasts, upper_lobe_of_left_lung, lung) Version 3.0. [27–30] Overlapping enhancers were fused. We included markers with a missing rate <10% or p-value for HWE >0.0001 or *heterozygosity excess* between −0.30 and +0.2. We also eliminated the marker with the lower MAF of a pair of common markers (MAF>0.01 and no more than 15,000 bp apart) with D´ larger than 0.8. In addition we left out imputed SNPs with low imputation accuracy, by r^2^<50% (reduced power), Iam_HWE_<50% (genotype information is less individual-specific than population-specific) or hiQ<50% (low inter-individual heterogeneity between imputed dosages). [31–33] We ended up with 52,207 SNPs included in the analysis.

### Statistical testing

First, we performed association tests for each marker individually using PLINK and RVTESTS, unadjusted and adjusted for 4 PCs (to infer genetic ancestry), sex and smoking status. The effect of a marker was modelled as log-additive. Since the onset of many rare and genetic diseases is at an early age, we fitted an additional model with a marker-age interaction.

In order to identify GARD genes associated with lung cancer, we performed gene-based tests compromising the whole set of markers assigned to a gene. We tested 1677 genes for multi-marker association with lung cancer, if required assuming an additive coding of genotypes. The following test/routines were applied: permutation test (implemented in PLINK [34]); ADD-SKATO test and all SNP ADD test (implemented in REGENIE [13]); Fp test, SKATO test and Zeggini test (implemented in RVTESTS [35])

We finally performed gene-set analyses for selected 620 HPO terms to identify those enriched with genes with low p-values for association with lung cancer, regardless of the statistical significance (p<0.05) of the contributing genes. [36] Kolmogorow-Smirnow tests (KS-tests) were performed. We highligted the Leading-Edge Subset (LES) of genes as the core of a gene set that accounts for the enrichment signal. [37]

### Level of significance

For the purpose of this study, p-values were considered continous indicators of statistical evidence. These were categorized according to the following scheme to account for multiple testing: The nominal significance level was set at *α* = 5%. The Bonferroni corrected level is given as *α*_*bon*_ = *α/k*, where k tests are performed in parallel. The suggestive level is given as *α*_*sug*_ = 1/*k*. Additional we consider a genome-wide level of significance of *α*_*g*.*w*_ = 1/*k*_*tot*_, where *k*_*tot*_ is the number of independent SNPs or genes in the genome.

When testing at the SNP-level, we set $\alpha_{g.w}^{\text{SNP}}=1\times 10^{-7}$, $\alpha_{\text{sug}}^{\text{SNP}}=2\times 10^{-6}$ (assuming k=500.000 [8]), and $\alpha_{\text{bon}}^{\text{SNP}}=8.5\times 10^{-7}$ (given 58,836 SNPs investigated).

When testing at the gene-level, we set $\alpha_{g.w}^{\text{gene}}=2.58\times 10^{-6}$, and $\alpha_{\text{sug}}^{\text{gene}}=0.0000515$ (assuming k=19.404 protein coding genes [26]), and $\alpha_{\text{bon}}^{\text{gene}}=0.0000298$ (given 1677 genes investigated).

When testing at the HPO-level (gene sets), we set $\alpha_{\text{sug}}^{HPO}=1.61\times 10^{-3}$, and $\alpha_{\text{bon}}^{HPO}=1.61\times 10^{-5}$ (given 620 HPO-terms investigated).

All data manipulations and analyses were performet with BCFTOOLS, VCFTOOLS, PLINK 1.90, RVTESTS, REGENIE an SAS^©^ 9.04.

## Results

### Case-Control series

The analysed series consists of 14,068 LC cases and 12,390 controls with median age of 63. Sixty-three percent were male, 52% of cases and 28% of controls were current smokers. The most frequent histological subtype is adenocarcinoma (38%), followed by squamous cell carcinoma (26%) and small cell lung cancer (10%).

### Single SNP association

**Figure 1:**
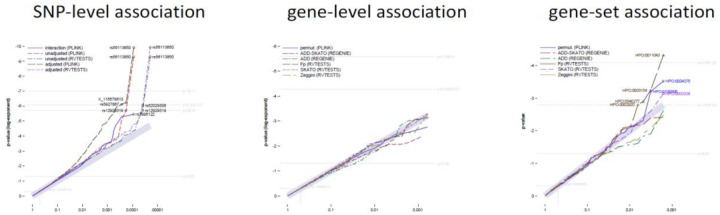
Comparative QQ-plots for the SNP-level association testing left panel: SNP-level association testing, central panel: gene-level association testing; right panel: gene-set (HPO-term) association testing; Footnote: levels of significance are defined as p=0.05 (local) p=0.0000515 (suggestive) p=2.58E-6 (genome-wide); most significant markers by test with FDR<0.05 are highlighted.

With a few exceptions, the p-values of PLINK and RVTESTS are sufficiently identical for the main effects (correlation of 0.99991 of the adjusted models). The corresponding QQ plot (figure 1, left panel) shows almost evenly distributed p-values across the markers, indicating a lack of significant associations.

A genome-wide significant association was observed only for rs56113850 (OR=0.893, 95%CI: 0.862–0.924, p=1.2×10^−10^, adjusted model), with a significant marker-age interaction (p=0.002). rs56113850 is located within the gene CYP2A6 (19q13.2).

For five other SNPs we observed suggestive significant associations. The p-values of theses SNPs are generally low across methods, indicating robustness of the observation. Due to the selection of markers for this analysis, this low number of significant associations had to be expected.

For the Duchenne muscular dystrophy gene *DMD* (rs1545663, rs5927867), a clear role in the pathogenesis of several cancers was reported. [38] The growth, migration and invasion of lung adenocarcinoma cells were slowed when Dp71, one of the shorter dystrophin protein variants, was switched off in vitro functional assays. [39] The other suggestive significant associations related to the *ABCC6* (rs12929319), *NDE1* (rs62029308) and *SLC614* (X_115578613) genes remain unexplained.

### Gene-based tests

Due to the selection of genes for this analysis, we could assume an almost uniform distribution of p-values, presented as QQ plot in figure 1 (central panel). However, we observed a skewed distribution for the p-values from REGENIE, and an unusual clustering of p-values>0.8 to then value 1 by PLINK and RVTESTS. Therefore, all p-values were corrected by logit adjustment, keeping their rank order of p-values. Details are contained in a supplement.

One to 637 genotyped markers were included in multimarker association tests per gene. On average, 10 markers were used by PLINK, 10.5 by RVTESTS and 11.5 by REGENIE. We did not find a single gene that was at least suggestively associated with LC in a multi-marker manner with any of the methods used.

**Figure 2:**
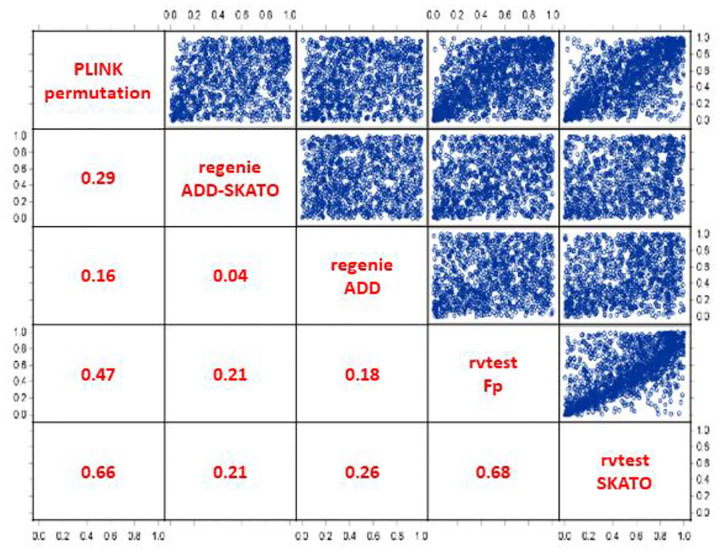
Spearmans Correlation of p-values between methods and routines

Apart from that, the correlation of the “corrected” p-values between the methods and routines was found low or moderate (between r=0.04 for REGENIE-ADD vs. -ADD-SKATO and r=0.68 for RVTESTS-Fp vs. –SKATO, see figure 2). This indicates a non-negligible influence of the respective multimarker association test used on the determination of “significance”. This inhomogeneity is partly due to deviating marker sets that were included in the respective analyses. PLINK e.g. cannot handle imputed SNPs.

### Gene-set analyses for selected 620 HPO terms

We also had to correct the p-values of the gene-set tests by logit adjustment, but found that the difference between derived and corrected p-values was negligible. Subsequently, no routine and no test method at the gene level generally yielded lower p-values than the others. In addition, the KS test used is based on the rank order and not on the p-values themselves, and is therefore robust to skewed values. The corresponding QQ diagram (figure 1, right panel) displays p-values that are almost uniformly distributed across all gene sets, with a few exceptions observed when using RVTESTS-Fp.

Within the 620 gene sets tested, the HPO-term HP:0011043 “Abnormal circulating adrenocorticotropin concentration” remained significant using the p-values of Fp-statistics after Bonferroni correction (p_corr._=4.7 ×10^−5^, p_uncorr._=4.3×10^−5^, RVTESTS-Fp). Only four of the 23 leading-edge genes (GMPPA, CYP11B1, POR, SMO) are themselves at least nominally significant (p<0.05). When using results from other gene-level tests, the p-values of this HPO-term ranged from 0.003 (RVTESTS -SKATO and -Zeggini) to 0.18 (REGENIE-ADD-SKATO).

Suggestively significant enrichment of associated genes was observerd for six further HPO-terms (4x based on p-values of RVTESTS, 2x based on PLINK results). Most p-values of these HPO-terms, when using results from other gene-level tests, were generally low (<0.25). We found in total 111 leading-edge genes overall seven HPO-terms (listed in Supplement **Error! Reference source not found.**). For 12 of them, the gene-level p-value were <0.05, as determined by at least one of the applied tests. (see Table 2) These 12 top genes can be divided into three categories of HPO terms. First, *BDNF, WDPCP, APC* and SMO are associated with neoplasms of the nervous system, especially the pituitary gland (group BN). Second, APC, ENO3, ACAD9, RNASEH1, FKTN, DYSF, and ATP2A1 are associated with Myalgia. Third, CYP11B1 and POR (other cytochrome P450 enzymes), GMPPA, and SMO are associated with the adrenocorticotropin (group ACTH).

### Functional gene network

Because mapping the interactions of proteins coded by associated genes is a key to understanding complex cellular mechanisms, we used *funcoup 6* [40, 41] to build human tissue-specific networks of all 111 leading-edge genes of the at least suggestive significant HPO terms. Multiple multi-omic data, collected from public online databases and resources used, are integrated with machine learning techniques by *funcoup 6* to represent interactomes. The tissue specificity for humans is based on protein expression extracted from Human Protein Atlas. [42] Networks show only genes with non-zero expression. We restricted the construction to the following tissues: lung, bronchus, nasopharynx, lymph nodes cerebral cortex, cerebellum, and hippocampus.

**Figure 3:**
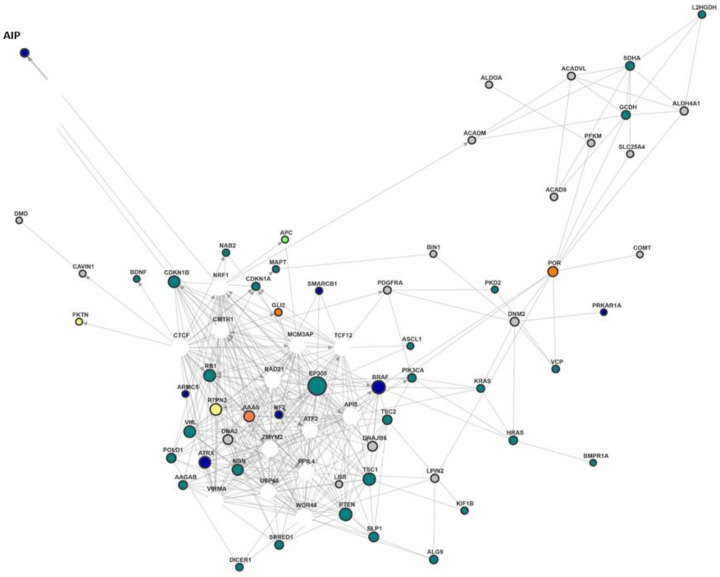
Gene expression network of LE-genes of at least suggestiveHPO-terms Gene expression network created by *funcoup 6*; only leading-edge genes of at least suggestive significant HPO-term are displayed; green dots: genes related to nervous system/pituitary gland; orange dots: genes related to ACTH circulation/level; blue dots: genes related to both the nervous system/pituitary gland and the ACTH cycle/concentration; grey dots: genes related to Myalgia, light/yellow dots: gene-level p-value<0.05; white dots: added by *funcoup 6* for building the network.

Although the list of leading-edge genes was compiled based on the association of DNA variants with LC, a compact but complex network of gene expressions was found (figure 3). The core of the network is mainly made up from leading-edge genes related to HPO-class BN (nervous system/pituitary gland; green dots). However, central in this core net one can find the genes *NF2* and *BRAF* (associated to HPO-class BN and ACTH), *EP300* (associated to HPO-class BN), *AAAS* (associated to HPO-class ACTH) and *PTPN2* (associated to Myalgia). The single gene-level p-values of these genes range from 0.03 to 0.27 (RVTESTS-Fp).

There is also a separate subnetwork that is only connected to the core net via the *POR* gene (cytochrome P450 oxidoreductase), a leading-edge gene of the association to Myalgia. Interestingly, the *POR* variation 503V was associated with faster *CYP2A6* activity (higher nicotine metabolite ratio). [43] It’s single gene-level p-value was 0.02 (RVTESTS-Fp). Only a few genes, such as *AIP* or *DMD* (discussed before), can be regarded as satellites outside the core network.

## Discussion

The International Lung Cancer Consortium (ILCCO) has compiled a large-scale genotype dataset of thousands of lung cancer cases and control subjects in a joint effort of several studies. With its help, numerous genomic loci associated with lung cancer risk have been detected. However, it can also be assumed that other as yet undiscovered genes are associated with lung cancer, especially if they are involved in complex molecular structures. We aimed to discover such genes and structures in focusing on rare diseases that affect the respiratory system.

By chance, we are able to re-detect the single-marker association of the marker rs56113850, which is located in the gene *CYP2A6* (19q13.2). This marker has already been associated to lung cancer in a joint GWAS of the ILCCO [24] and is considered primarily informative for LC prediction. [12] Therefore, rs56113850 was initially left out from this analysis but included again in the group of analysed markers as it is located in an enhancer region for the GARD gene LTBP4 (e.g. associated with cutis laxa with severe lung abnormalities, ORPHA code 221145, *OMIM* no. 613177). In most smokers, *CYP2A6*-catalyzed C-oxidation accounts for >75% of nicotine metabolism. The activity of this major nicotine-metabolizing enzyme has been shown to correlate with the amount of nicotine and carcinogens drawn from cigarettes. [43, 44] *CYP2A6* polymorphisms are well known to alter tobacco-related cancer risks. [45, 46] Smoking intensity accounted for 82.3% of the effect of *CYP2A6* activity on lung cancer risk but entirely mediated the genetic effect of rs56113850. [47]

However, the pleiotropy of *CYP2A6* and *LTBP4* may be the molecular reason why tobacco smoking can lead to a rapid, severe loss of lung function in individuals with *LTBP4*-related cutis laxa. [48, 49]

Two further markers (rs1545663, rs5927867) are associated with LC with suggestive significance. These can be assigned to the Duchenne muscular dystrophy gene *DMD*. A clear role in the pathogenesis of various types of cancer has been reported for this gene. [38] Furthermore, the growth, migration and invasion of lung adenocarcinoma cells were slowed when Dp71, one of the shorter dystrophin protein variants, was switched off in vitro functional assays. [39]

Although being unable to prove any single GARD gene to be associated with LC, a significant enrichment of genes involved in an abnormal circulating adrenocorticotropic concentration (three HPO-Terms) was observed. The adrenocorticotropic hormone (ACTH) is mainly produced by the anterior pituitary gland. Via the Hypothalamic-Pituitary-Adrenal (HPA) axis, it stimulates the production and release of cortisol. The HPA system hence influences the central nervous system and the endocrine system by regulating the balance of hormones in response to stress. Tumours outside the pituitary gland are known to also produce ACTH; in particular, a large proportion of SCLC (around 85–90%) expresses neuroendocrine biomarkers such as adrenocorticotropic hormone (ACTH). [50]

Nicotine is a strong activator of the hypothalamus pituitary adrenal (HPA) axis. Smoking of only two cigarettes consistently activates the HPA axis of habitual smokers. On the other hand, chronic inflammation of the airways is a common consequence of habitual smoking, and smokers often present with low-grade systemic inflammation, which may be mediated by HPA axis alterations. The main effective component of cigarette smoke on the HPA axis seems to be nicotine. [51] Therefore, it does not seem surprising that some genes that are more or less directly involved in the regulation of the HPA axis show a low association with lung cancer. We could not identify an explicit functional pathway that should be considered relevant for lung cancer risk. However, we found that most of the leading genes of our analysis that are related to the HPA axis interact in a complex manner. It seems unlikely that such weak or entangled associations can make a useful contribution to risk assessment with regard to lung cancer.

All in all, one should not expect to be able to uncover any unrecognised associated genes through tests at the gene level, but rather through pathway analyses. The results of the gene-level tests were found to be extremely inhomogeneous with partially distorted p-values, even when applied to the same data.

## Conclusions

Genes associated with genetic and rare lung diseases (GARD) do not appear to be risk factors for lung cancer. However, genes related to the hypothalamic-pituitary-adrenal (HPA) axis show some, but rather weak or intricate associations with lung cancer. Tests at the gene level provide extremely inhomogeneous results, even when applied to the same data.

## Supplementary Files

This is a list of supplementary files associated with this preprint. Click to download.

- GARDgenesandLCSupportinginformationV1.8ohneKorrekturen.pdf

## Figures and Tables

**Table 1:** At least suggestive significant HPO terms

HPO class	HPO no.	test applied	p_corr_^†^	# LE genes(# p<0.05)	HPO term
		**Bonferroni correction: *p*** ≤ **1.61×10^−5^**			
ACTH	HPO:0011043	RVTESTS -Fp	4.7 ×10^−5^	23 (4)	Abnormal circulating ACTH concentration
		**Suggestive significance: *p*** ≤ **1.61×10^−2^**			
BN	HPO:0004375	PLINK-permutation	0.0003	53 (3)	Neoplasm of the nervous system
ACTH	HPO:0003154	RVTESTS-Fp	0.0006	13 (3)	Increased circulating ACTH level
BN	HPO:0100006	PLINK-permutation	0.0006	44 (3)	Neoplasm of the central nervous system
Myalgia	HPO:0003326	RVTESTS-SKATO	0.0007	57 (7)	Myalgia
BN	HPO:0040277	RVTESTS -Fp	0.0010	25 (2)	Neoplasm of the pituitary gland
ACTH	HPO:0002920	RVTESTS -Fp	0.0020	12 (1)	Decreased circulating ACTH concentration

p_corr_^†^ corrected p-values of a Kolmogorow-Smirnow test for gene-sets; ŧ p-value according to the listed applied test; ACTH adrenocorticotropin hormone; BN brain/nervours system; LE leading-edge, HPO Human Phenotype Ontology

**Table 2:** Significant (p_min_<0.05) LE genes in at least suggestive significant HPO terms

GENE	MINIMAL P-VALUE^†^	TEST	CLASS 1	2	3	4/5	HPO NO.	-	-	-	-
***BDNF***	0.03	RVTESTS-Zeggini	BN	BN			HPO:0004375	HPO:0100006			
***MAPT***	0.02	RVTESTS-SKATO	BN	BN			HPO:0004375	HPO:0100006			
***APC***	0.005	RVTESTS-Fp	Myalgia	BN	BN	BN	HPO:0003326	HPO:0004375	HPO:0040277	HPO:0100006	
***SMO***	0.02	RVTESTS-Fp	ACTH	BN	ACTH	BN	HPO:0002920	HPO:0004375	HPO:0011043	HPO:0040277	HPO:0100006
***BRAF***	0.02	RVTESTS-Zeggini	ACTH	ACTH	BN		HPO:0003154	HPO:0011043	HPO:0040277		
***CYP11B1***	0.005	PLINK-permutation	ACTH	ACTH			HPO:0003154	HPO:0011043			
***GMPPA***	0.01	RVTESTS-Fp	ACTH	ACTH			HPO:0003154	HPO:0011043			
***POR***	0.02	RVTESTS-Fp	ACTH	ACTH			HPO:0003154	HPO:0011043			
***ENO3***	0.003	RVTESTS-Zeggini	Myalgia				HPO:0003326				
***LPIN2***	0.01	REGENIE-ADD	Myalgia				HPO:0003326				
***ACAD9***	0.02	RVTESTS-SKATO	Myalgia				HPO:0003326				
***FKTN***	0.02	RVTESTS-Fp	Myalgia				HPO:0003326				
***RNASEH1***	0.02	RVTESTS-SKATO	Myalgia				HPO:0003326				
***ACP5***	0.03	REGENIE-SKATO	Myalgia				HPO:0003326				
***DMD***	0.03	PLINK-permutation	Myalgia				HPO:0003326				
***PTPN2***	0.03	RVTESTS-Fp	Myalgia				HPO:0003326				
***ATP2A1***	0.04	PLINK-permutation	Myalgia				HPO:0003326				
***CAPN3***	0.04	REGENIE-ADD	Myalgia				HPO:0003326				
***DYSF***	0.04	RVTESTS-SKATO	Myalgia				HPO:0003326				

† minimal p-value of all gene-level tests applied; HPO Human Phenotype Ontology

## Data Availability

The case-control data that support the findings of this study are available from ILCCO/INTEGRAL from the authors upon reasonable request and with permission from the ILCCO/INTEGRAL data access committee. The following publicly available datasets were used in this work: Prostate, Lung, Colorectal, and Ovarian Cancer Screening Trial, phs000093.v2.p2; FLCCA study, phs000716.v1.p1; EAGLE study, phs000336.v1.p1; German, SLRI, IARC, and MD Anderson Cancer Center studies, phs000876.v2.p1; Oncoarray study, phs001273.v3.p2; imputed Oncoarray study using HRC reference panel, phs001273.v4.p2;
